# A Magnetic Field-Viewing Film-Based Probe for Imaging and Quantitative Evaluation of Hidden Corrosion in Coated Ferromagnetic Conductors

**DOI:** 10.3390/mi17050529

**Published:** 2026-04-26

**Authors:** Bei Yan, Xiaozhou Lü, Chengming Xue, Yong Li

**Affiliations:** 1 School of Aerospace Science and Technology, Xidian University, Xi’an 710071, China; yanbei@xidian.edu.cn (B.Y.); xuechengming@xidian.edu.cn (C.X.); 2 State Key Laboratory for Strength and Vibration of Mechanical Structures, Shaanxi Engineering Research Centre of NDT and Structural Integrity Evaluation, School of Aerospace Engineering, Xi’an Jiaotong University, Xi’an 710049, China; yong.li@mail.xjtu.edu.cn

**Keywords:** magnetic field-viewing film, coated ferromagnetic conductor, hidden corrosion, opening profile identification, quantitative evaluation

## Abstract

Coated ferromagnetic conductors (CFCs) are widely used in the engineering field, such as transportation, petrochemicals, energy, etc. Owing to long-term exposure to harsh and corrosive environments, involving large temperature differences, cyclic loading and humidity, hidden corrosion occurring under the coatings of CFCs has been found to be one of the most critical defects posing a severe threat to the structural strength and safety of CFCs. Therefore, it is important to conduct rapid imaging and quantitative evaluation of this hidden corrosion via Non-Destructive Evaluation (NDE) techniques. A magnetic field-viewing film (MFVF) characterizes magnetic fields by displaying corresponding color shifts, offering a direct visual representation of the magnetic field intensity. In light of this, this paper proposes an MFVF-based probe composed of multiple micro-sensor units for fast imaging of hidden corrosion in CFCs. An image-processing technique based on the modified Canny algorithm is subsequently proposed for identification of corrosion opening profiles in MFVF images. Based on the identification results, an assessment of hidden corrosion parameters is conducted. It is inferred from the experimental results that the opening area, depth and volume of hidden corrosion can be quantitatively evaluated, with an average accuracy of 86.1%.

## 1. Introduction

Coated ferromagnetic conductors (CFCs), consisting of a non-conductive coating and ferromagnetic substrate, are extensively utilized in engineering fields, such as transportation, petrochemicals, energy, etc., by virtue of their exceptional characteristics of high strength and low cost [1,2,3]. Nevertheless, due to hostile environments, involving large temperature differences, cyclic loading and humidity, defects, particularly corrosion occurring under the coating of an in-service CFC, have been found to pose a severe threat to structural strength and safety. Therefore, Non-Destructive Evaluation (NDE) techniques are in high demand for defect identification and quantitative evaluation and yield significant engineering value. For defect evaluation, NDE techniques including Eddy Current Testing (ECT) [4,5], Ultrasonic Testing (UT) [6,7], Infrared Testing (IRT) [8,9], Penetrant Testing (PT) [10], Magnetic Particle Testing (MPT) [11], Magneto-Optical Imaging (MOI) [12,13] and Magnetic Flux Leakage (MFL) [14,15] are usually employed for inspection of CFCs.

With the proposition of Non-Destructive Testing (NDT) 4.0 based on Industry 4.0, visual inspection has emerged as a pivotal trend in NDT [16,17]. Consequently, much research focused on advanced techniques for testing signal processing and defect imaging has been carried out. Shao et al. processed the signals obtained from a hand-held ECT multi-channel sensor array, consisting of sixteen coils, via Hough Circle Transformation and texture analysis in a bid to achieve real-time visual inspection of defects in riveted joints [18]. Ding et al. proposed an eddy current array, consisting of 48 coils, with a diameter of 2 mm, arranged in three rows, with 16 coils per row, for defect images in finely grooved structures [19]. Zhu et al. proposed an ultrasonic super-resolution imaging model based on up-sampling and down-sampling layers and multi-layer residual networks to improve the resolution of ultrasonic images [20]. Yuan et al. presented a 3D visual reconstruction method for evaluating corrosion defects using the alternating-current field measurement (ACFM) technique, which was verified through experiments. However, the ACFM probe was equipped with only one magnetic field sensor, requiring 25,921 scans, with a step size of 0.5 mm over an 80 mm × 80 mm rectangular region, to acquire an image of a circular defect with a diameter of 30 mm [21]. Joan Stephanie G. Elizalde provides a simple approach to detecting and determining hidden corrosion using active infrared thermography, which involves imaging the specimen with an infrared camera after heating it for more than 10 min [22]. Wu et al. investigated an improved post-processing method for infrared images based on temperature image division for improving the signal-to-noise ratio of images and increasing the detection rate of defects [23]. Ou et al. reported a vision-based quality evaluation method for evaluating the quality of the PT process and producing outputs applicable to an automated PT control system [24]. Li et al. presented a defect detection algorithm for reducing the likelihood of false negatives by applying a method for bearing image defect detection that combines magnetic particle inspection with deep learning [25]. Murshudov et al. developed a software approach for optimizing the spacing between magnetic sensors used for MFL imaging, with the goal of determining both the minimum number of sensors required in the array and the optimal spacing [26]. Marsic et al. proposed an MFL probe, consisting of 10 hall sensors, with a spacing of 5 mm, for scanning a steel sample, with a step size of 1 mm, to evaluate the thickness of a steel plate [27]. Ma et al. put forward MOI under a vertical combined magnetic field to obtain magneto-optical images of radial cracks in spot welds, with an area of 4 mm × 4 mm [28].

In spite of the aforementioned technical advancements, when applied to industry fields, visual inspection technologies still have the problem of low detection efficiency, e.g., (1) due to factors such as the power, circuitry, size and weight of the testing equipment, the number of sensors in the sensor array of either the ECT, ACFM, UT or MFL system is limited, resulting in relatively low detection efficiency and a relatively low spatial resolution of the defect images; (2) the preparation work for MPT or PT is normally time-consuming, leading to low detection efficiency; (3) IRT requires extra heat sources to heat the specimen for a relatively long time before obtaining the IRT image and sometimes even needs to acquire the IRT image during the cooling process, thus resulting in low detection efficiency; and (4) the sophisticated MOI system limits the MOI detection area to a narrow region, thereby causing low detection efficiency.

It is noticeable that the development in magnetic-field imaging technology holds promise for opening a door to new approaches for efficient and high-resolution evaluation of hidden corrosion in CFCs. Hua et al. demonstrated the imaging of complex magnetic fields by using the magneto-photonic effect of an Fe_3_O_4_@SiO_2_ nanorod suspension with one-to-one correspondence between visible colors and magnetic field directions [29]. Markoulakis et al. encapsulated a micron-thin film of ferrofluid in a vacuum inside a lens and applied it to a custom-made optical microscope for the observation and research of dynamic magnetic fields [30]. Yang et al. proposed a method for the simultaneous imaging of static and microwave magnetic field distributions using magneto-optical indicator microscopy [31].

In view of the technological development of magnetic field imaging, this paper innovatively combines a magnetic field-viewing film (MFVF) with permanent magnets and proposes an MFVF-based probe. The probe requires no power source and is applicable to high-efficiency hidden corrosion imaging with relatively simple operations. Compared with existing NDT technologies, the MFVF-based technology has advantages, such as an ultra-high spatial resolution, a large detection area, high detection efficiency, and low cost. An inspection system for direct imaging and quantitative evaluation of hidden corrosion was built. The feasibility of the proposed NDT technique for efficient assessment of the hidden corrosion in a CFC was intensively investigated through experiments.

The rest of this paper is organized as follows: Section 2 elaborates on the basic principles of a magnetic field-viewing film (MFVF) and proposes an MFVF-based probe for detecting hidden corrosion efficiently, combined with simulation analysis. In Section 3, the experimental system with an MFVF-based probe for inspection of hidden corrosion in a CFC is presented, followed by the proposition of the modified Canny algorithm based on an improved small sub-domain filtering method for opening-profile identification of the hidden corrosion. The evaluation method for estimation of the corrosion depth and volume based on the corrosion opening profile is proposed and investigated in Section 4.

## 2. A Magnetic Field-Viewing Film-Based Probe

**Figure 1:**
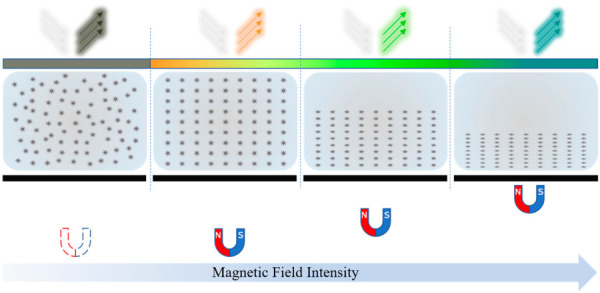
Working principle of MFVF.

Depending on the different principles of sensitivity to magnetic fields, magnetic field-sensing films come in various types. A ferromagnetic thin film coated with fluorescent dyes generates heat in low-frequency magnetic fields, causing the fluorescent layer to change color, and magnetic field mapping can be obtained in a short time and on a large surface by recording images with an S-CMOS camera [32,33]. An optical indicator made of a bismuth-substituted yttrium iron garnet thin film coated with a metal thin film layer was used to simultaneously visualize static and microwave magnetic fields for a 1 mm × 1 mm area, with sensitivities of approximately 0.1 and 1.1 Gauss, respectively [34]. A ferrolens, consisting of two optical-quality glass disks separated by a thin film of superparamagnetic fluid, can display the discrete magnetic flux lines of a macroscopic field [35]. A poly-dimethylsiloxane (PDMS) film sealed with Fe_3_O_4_@SiO_2_ nanorod suspensions was used to visualize complex magnetic fields by utilizing the magneto-photonic effect of an Fe_3_O_4_@SiO_2_ nanorod suspension with one-to-one correspondence between the visible colors and magnetic field directions [29]. Taking into account the electromagnetic properties of a CFC and the structural characteristics of hidden corrosion, in this study, an MFVF yielding the direct imaging of a magnetic field, with the color closely associated with the field intensity, was employed for the fast imaging of hidden corrosion in a CFC. The basic principle of the MFVF is shown in Figure 1.

As presented in Figure 1, the ferromagnetic nanoparticles are randomly distributed inside the MFVF. In the absence of an external magnetic field, the microstructure of an MFVF absorbs the light of all colors except for dark olive green and, thus, reflects dark-olive-green light. This gives rise to the color of the MFVF of dark olive green. When the MFVF is subject to a magnetic field with a varying intensity, the nanoparticles are reorganized into a chainlike structure, whilst they are aligned in the magnetic field’s direction. The nanoparticle reorganization, consequently, alters the MFVF’s microstructure, resulting in a significant color shift in the MFVF due to the change in the wavelength of the reflected light from the film. Therefore, it is straightforward to directly capture the magnetic field distribution within the spatial area where the MFVF is deployed through the intrinsic relation between the magnetic field intensity and the MFVF color.

**Figure 2:**
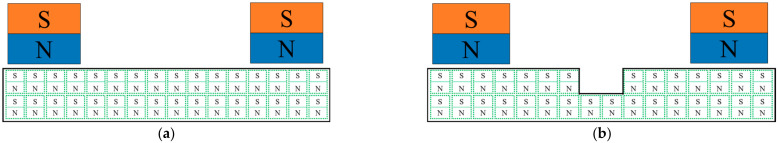
Working principle of MFVF-based probe: (**a**) CFC without corrosion; (**b**) CFC with corrosion.

It is noted that a single nanoparticle is a magnetic field-sensing unit. Therefore, the MFVF can be regarded as a magnetic field sensor with an ultra-high spatial resolution. Thanks to the MFVF’s characteristics, an MFVF-based probe containing a sheet of the MFVF with two parallel permanent magnets on it is capable of efficiently visualizing hidden corrosion in CFCs. The working principle of the MFVF-based probe is portrayed in Figure 2. A CFC magnetized by permanent magnets can be considered to be composed of multiple small permanent magnets aligned in the same direction. The small permanent magnets in the corrosion-free area are arranged regularly; thus, the magnetic field barely has abrupt changes. In the corrosion area, material loss leads to the absence of some small permanent magnets, resulting in a significant change in the magnetic field. The magnetic field change can be sensed by the MFVF-based probe for acquisition of an MFVF image for hidden corrosion evaluation.

**Figure 3:**
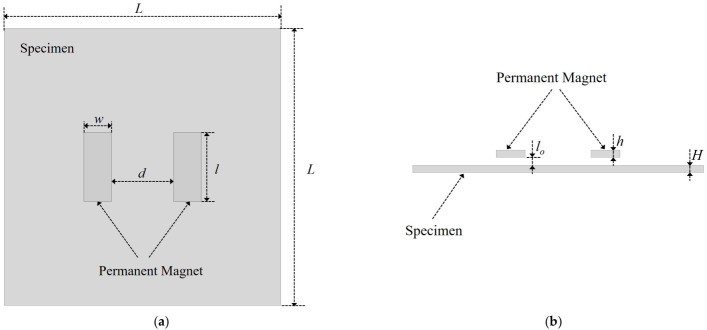
A corrosion-free finite element model: (**a**) top view; (**b**) front view.

Subsequently, finite element analysis was employed to verify the feasibility of the MFVF-based probe for evaluating hidden corrosion in a CFC. Given the positive correlation between MFVF color and magnetic field intensity, if there is a distortion in the magnetic field intensity at the location of the hidden corrosion in the region between the two permanent magnets, this indicates that the MFVF-based probe has the capability to detect hidden corrosion. A corrosion-free finite element model is shown in Figure 3. The model consists of two permanent magnets and a specimen. It is worth noting that for a finite element model, the electromagnetic parameters of the non-ferromagnetic coating are identical to those of air. Therefore, the surface of the component covered with a coating of thickness lo can be simulated by setting the lift-off *l*_o_.

**Figure 4:**
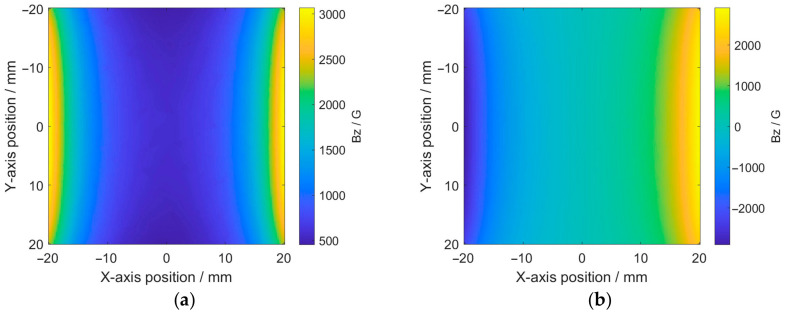
Distribution of magnetic field intensity in detection area: (**a**) same pole directions; (**b**) opposite pole directions.

The material of the specimen was Q235 steel, with a relative permeability of 300. The grade of the permanent magnets was N52. The main geometric parameters of the finite element model are shown in Table 1. The region between the two permanent magnets was considered the detection area. Simulations were conducted separately for the cases where the magnetic field directions of the two permanent magnets were the same and opposite, with the distribution of the magnetic field intensity in the detection area shown in Figure 4.

It can be seen from Figure 4 that when the magnetic poles are the same, the magnetic field distribution within the detection area is relatively uniform and exhibits no directional change. Conversely, when the magnetic pole directions are opposite, the magnetic field distribution shows significant differences, with a sudden directional change occurring at the center of the detection area. Therefore, the magnetic pole direction of the magnet in the probe should be the same, and the S poles of the two permanent magnets can simultaneously be perpendicular to the MFVF, either upward or downward.

**Figure 5:**
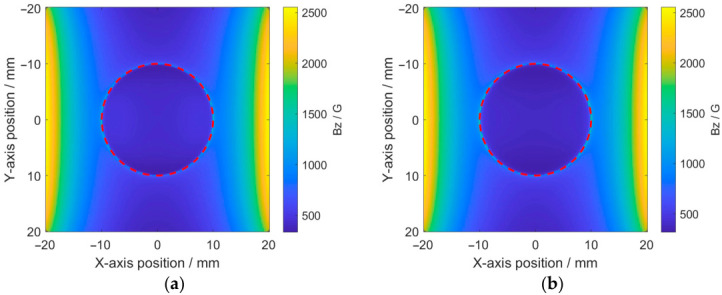
Distribution of magnetic field intensity in detection area: (**a**) corrosion with a depth of 1 mm; (**b**) corrosion with a depth of 2 mm.

After determining the magnetic pole direction of the magnets, the feasibility of the MFVF-based probe for detecting hidden corrosion was further verified. Simulations were conducted using circular-shaped corrosion. with a diameter of 20 mm and depths of 1 mm and 2 mm, respectively. The results are shown in Figure 5.

**Figure 6:**
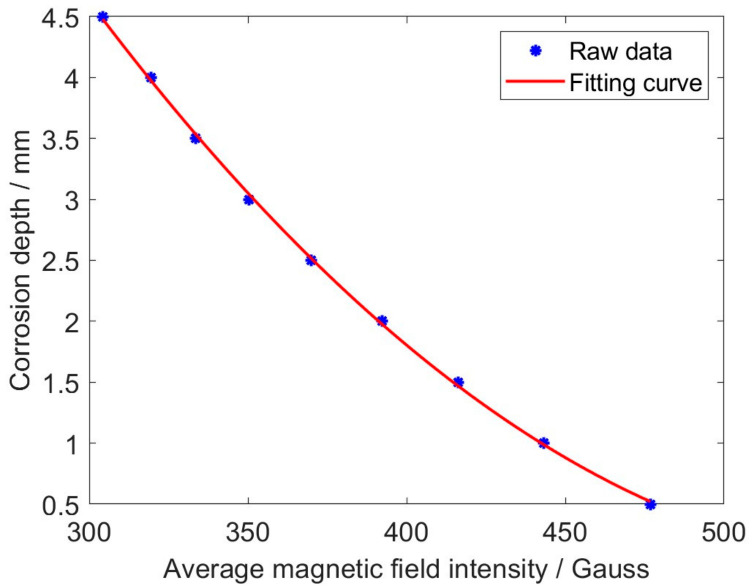
The average magnetic field intensities vs. corrosion depths.

It can be observed from Figure 5 that the magnetic field intensity varies abruptly along the boundary of the corrosion opening (indicated by red dashed lines), implying that the hidden corrosion can be identified through MFVF images. It is evident that there is a difference in the magnetic field intensity between the corroded region and the non-corroded region, and this difference increases with greater corrosion depth. To further analyze the relationship between magnetic field intensities and corrosion depths, simulations were conducted on a series of 20 mm diameter circular-shaped corrosion regions with varying depths. The average magnetic field intensities within the corrosion regions were calculated, and the results are shown in Figure 6.

It is noticeable from Figure 6 that the average magnetic field intensity decreases as the corrosion depth increases and follows a quadratic function variation pattern. By taking the quadratic fitting of the average magnetic field intensities, a fitting curve in the form of a quadratic function is obtained. The function corresponding to the fitting curve is formulated as

(1) $$d=0.00006505a^{2}-0.0737a+20.8952$$

where *a* denotes the average magnetic field intensity, whilst *d* is the corrosion depth.

The simulated raw data show a good fit with the fitted curve, with a maximum relative error of 1.4%. The simulation results demonstrate that quantitative corrosion assessment can be realized by accurately identifying the corrosion profile and calculating the average magnetic field intensity in the corrosion region. Given the existence of an associative relationship between magnetic field intensities and MFVF colors, quantitative corrosion assessment can be conducted by utilizing experimentally acquired MFVF images.

## 3. Experiments and Image Processing

### 3.1. The Inspection System

**Figure 7:**
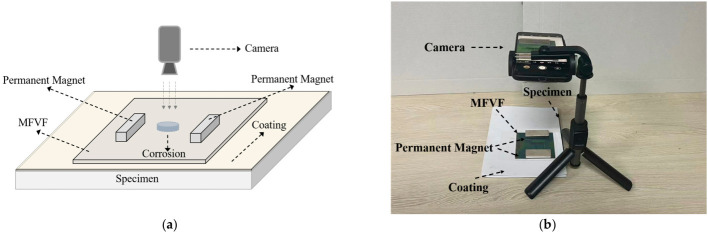
The inspection system: (**a**) schematic illustration; (**b**) practical picture.

The schematic illustration and practical picture of the established inspection system are portrayed in Figure 7, respectively. The system consisted of (1) a pair of permanent magnets (individually with a size of 50 mm × 20 mm × 5 mm in length × width × thickness) for locally magnetizing the corroded area of the specimen; (2) the MFVF (NANOBRICK MX-Film) for visualizing the distribution of the magnetic field; and (3) an embedded camera of a mobile phone (Mate 70, Huawei Technologies Co., Ltd., Shenzhen, China) for capturing raw testing images (the time for taking the photo was less than 0.1 s).

It is noteworthy that (1) the MFVF was encapsulated to enhance its strength, and, thus, the thickness of the MFVF was increased from 0.2 mm to 0.5 mm; (2) the distance between the two magnets was 45 mm; (3) the vertical distance between the camera and the specimen was 180 mm; and (4) multiple sheets of paper (with a total thickness of 0.5 mm) were attached to the surface of the fabricated specimen for simulating non-magnetic coating materials such as paint, rubber, plastic, and aluminum.

Since the effective extraction of the corrosion information from the acquired MFVF images highly relies on the correlation of the magnetic field intensity with the MFVF color, the correlation between the MFVF color and the intensity of the magnetic field imposed on the MFVF is analyzed and listed in Table 2.

### 3.2. Testing Specimen

**Figure 8:**
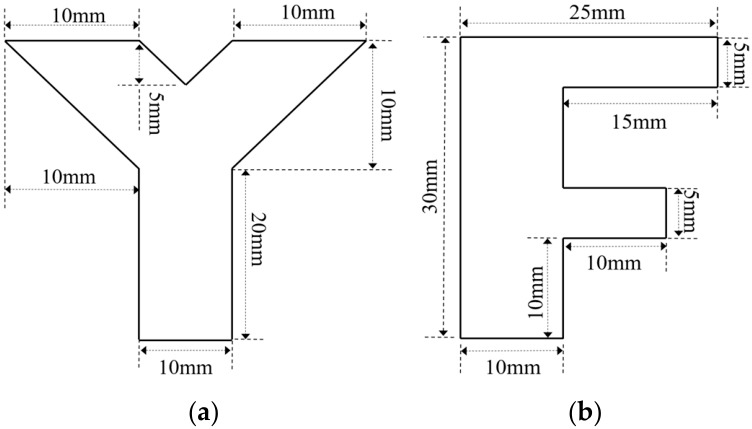
Schematic illustration of irregular-shaped corrosion: (**a**) Corrosion #Y; (**b**) Corrosion #F.

In order to simulate natural hidden corrosion, corrosion with different opening profiles and depths was fabricated on the surfaces of carbon steel plates (with a thickness of 5 mm) [36]. The fabricated corrosion on the specimens included (1) circle-shaped corrosion, with the diameter fixed at 10 mm and depths of 1 mm (Corrosion #1), 2 mm (Corrosion #2), 3 mm (Corrosion #3) and 4 mm (Corrosion #4); (2) square-shaped corrosion, with the side length fixed at 10 mm and depths of 1 mm (Corrosion #5), 2 mm (Corrosion #6), 3 mm (Corrosion #7) and 4 mm (Corrosion #8); and (3) irregular-shaped corrosion, including Corrosion #Y and Corrosion #F, with the depths of 3 mm. The shapes and dimensions of Corrosion #Y and Corrosion #F are presented in Figure 8.

### 3.3. Imaging of the Hidden Corrosion

**Figure 9:**
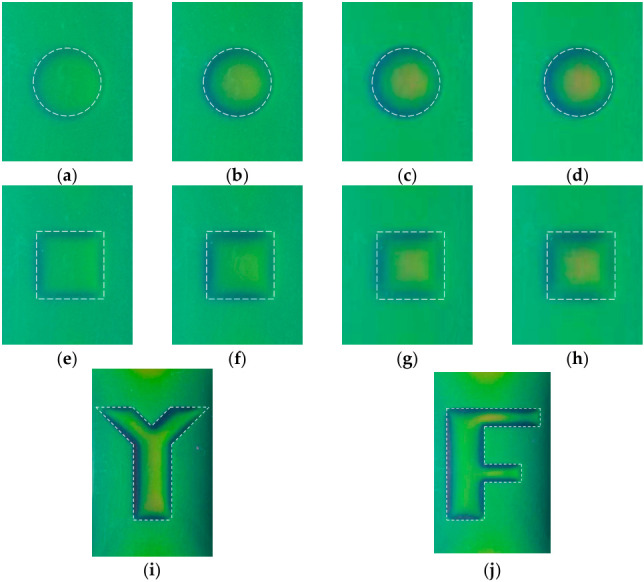
Raw MFVF images of hidden corrosion: (**a**) Corrosion #1; (**b**) Corrosion #2; (**c**) Corrosion #3; (**d**) Corrosion #4; (**e**) Corrosion #5; (**f**) Corrosion #6; (**g**) Corrosion #7; (**h**) Corrosion #8; (**i**) Corrosion #Y; and (**j**) Corrosion #F.

During the experiments, the camera was aligned perpendicularly to the corrosion area, and the environmental lighting was constant to ensure the acquisition of consistent and standardized raw images. The acquired raw corrosion images are shown in Figure 9, together with the dashed line, indicating the true corrosion opening profile.

As can be observed in Figure 9, the MFVF color of green corresponds to the corrosion-free region, while the MFVF color varies abruptly along the corrosion edge. For a series of corrosions with the same opening profile but different depths, there are differences in the MFVF color within the corroded areas. When the corrosion depth increases, the MFVF color tends to be red. This indicates that it could be possible to assess the corrosion depth based on the color of the MFVF, particularly in the corrosion region, which can be recognized intuitively from the raw images. It is worth noting that the resolution of the raw images is 150 DPI, which corresponds to 150 × 150 magnetic field sensors within 1 in^2^, demonstrating the inherent advantage of the MFVF-based probe in terms of its high spatial resolution.

**Figure 10:**
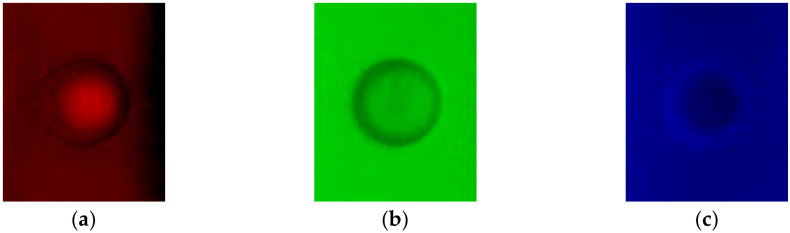
Three channels of raw image for Corrosion #4: (**a**) red channel; (**b**) green channel; and (**c**) blue channel.

It is noticed that the corrosion profile identification is pivotal for quantitatively evaluating the detected corrosion. In an effort to improve the accuracy of the corrosion profile identification, the signal-to-noise ratio (SNR) of the raw corrosion images has to be enhanced via image processing. In a bid to analyze the characteristics of a corrosion image, by taking Corrosion #4 as an example, the raw image was fed into the red channel, green channel and blue channel. The processed images are portrayed in Figure 10.

It is noticeable from Figure 10 that (1) the red-channel and blue-channel images have relatively blurry gradients at the corrosion edges and, thus, are hardly applicable for the identification of corrosion edges; (2) the raw images contain small spots with random variations in brightness and color. Further analysis reveals that they have a Gaussian distribution, making the images blurry and grainy with the loss of corrosion detail and unsharp edges. In light of this, the standard deviation of the images in Figure 7 is calculated by using the expression written as

(2) $$\sigma_{d}=\sqrt{\frac{1}{n}\sum_{i=1}^{n}{\left(x_{i}-\frac{1}{n}\sum_{i=1}^{n}x_{i}\right)}^{2}}$$

where *σ_d_* is the standard deviation. *x* denotes the value of each pixel, whilst *n* represents the number of pixels. By using Equation (2), the standard deviation of a 5 mm × 5 mm (*n* = 872) square region in the corrosion-free area is computed. The results are listed in Table 3.

From Table 3, it can be seen that the green channel gives the smallest standard deviation, indicating that the green-channel image has the highest SNR. Therefore, the subsequent investigation is focused on the processed images via the green channel of the raw corrosion images for quantitative evaluation of each corrosion.

### 3.4. Opening Profile Identification of Hidden Corrosion

The opening profile is the basic feature of MFVF images, and it contains the location and size of hidden corrosion and plays an important role in further quantitative analysis of hidden corrosion. As a popular edge detection algorithm developed by John F., the Canny algorithm is widely applied in image processing [37]. The traditional Canny algorithm employs a filter based on the derivative of a Gaussian distribution for computing the gradient intensity, whilst Gauss filtering denoises the image. Through this processing, the potential edge is converged to a one-pixel-wide line by eliminating non-maximum pixels of the gradient intensity. The edge pixels are kept or removed via the double-threshold processing to determine edges.

**Figure 11:**
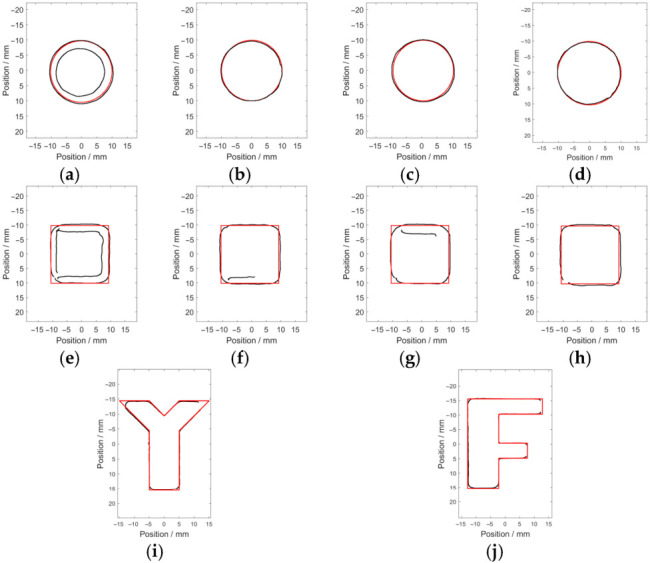
The identified opening profiles of the hidden corrosion via the Canny algorithm: (**a**) Corrosion #1; (**b**) Corrosion #2; (**c**) Corrosion #3; (**d**) Corrosion #4; (**e**) Corrosion #5; (**f**) Corrosion #6; (**g**) Corrosion #7; (**h**) Corrosion #8; (**i**) Corrosion #Y; and (**j**) Corrosion #F.

The Canny algorithm was used for identifying the opening profile of the green channel of the raw corrosion images, with the main parameters of the width of the Gaussian filter, the maximum value, and the minimum value being 5, 0.4 and 0.8 respectively. The identified edge pixels (indicated by the black solid line) of the hidden corrosion are shown in Figure 11.

It is observed from Figure 11 that the corrosion opening profile can be roughly identified. However, due to the existence of spurious edge pixels and the loss of true edge pixels, apart from Corrosion #2, Corrosion #3 and Corrosion #4, it is difficult to identify the true opening profile of the other corrosion from the image.

In order to enhance the contrast of the image on the opposite side of the corrosion edge in the green-channel image and improve the accuracy regarding the corrosion edge detection, a modified Canny algorithm is proposed. The procedure of the corrosion edge identification includes the following:

(1) Since the spurious edges introduced by noise reduce the accuracy of the edge detection, Gaussian filtering for a smoothing process is, thus, used for image denoising. The Gaussian filtering smooths an image by convolving it with a Gaussian kernel, effectively suppressing the high-frequency noise while maintaining the sharpness of edges. The Gaussian kernel can be written as
  (3) $$G\left(m,n\right)=\frac{e^{-\frac{m^{2}+n^{2}}{2\sigma^{2}}}}{2\pi \sigma^{2}}$$
  where *m* and *n* are the spatial coordinates of the kernel. *σ* is the width of the Gauss filter, which directly controls the effect of filtering. The Gaussian kernel slides over all pixel points in the image and processes all pixel points by taking a weighted average value of neighboring pixel points.

(2) After the denoising of the image, the traditional Canny algorithm computes the magnitude and direction of the gradient for each pixel in the image with the convolution operators [38]. Although calculating image gradients using convolution operators is efficient, it has drawbacks due to the fact that (1) it is only sensitive to edges in specific directions and can rarely respond to edges in all directions; (2) since the gray-level variations arising from the noise and those caused by real edges exhibit similar local characteristics, the convolution operators can hardly distinguish the noise from real edges, leading to the misjudgment of the noise-induced gray-level changes as real edges; and (3) the asymmetry of gradient operators is prone to edge offset. In view of this, an improved Small Sub-Domain Filtering Method (SSFM) is proposed to replace the gradient operator, which is also a key step of the modified Canny algorithm.

The SSFM is an effective approach to defining the edges of sources, and it is usually applied to outline the horizontal edges of gravity and magnetic sources [39]. It performs low-pass filtering on the data based on the moving window sub-domain averaging principle for enhancing image contrast. For every scanning position, the average standard deviation in the pixel-enclosed sub-domain filter window is calculated. The average of the minimum standard deviations of the entire scanning positions of the sub-domain filter window is used instead of the pixel value at the reference point.

**Figure 12:**
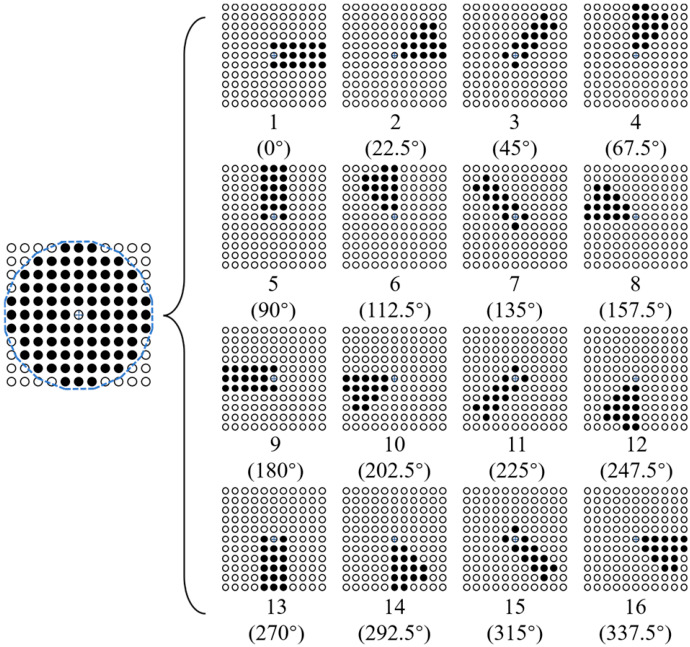
The schematic illustration of the modified SSFM.

To apply the SSFM to preprocessing raw MFVF images, it is noted that (1) the divided sub-domains need to have symmetry to prevent gradient offset of the image data; (2) as many sub-domains in different directions as possible should be divided to prevent local anomalies in the data; and (3) the scanning trajectory should be close to a circle to prevent data distortion. In light of this, an improved SSFM is proposed, and the schematic illustration of the modified SSFM is elaborated in Figure 12. The pixels contained in the sub-domains are expressed as Equation (4).

(4) $$\left\{\begin{array}{l} \sigma_{1}=[\underset{k=n,n+1,\ldots ,n+5}{\underset{︸}{f(m-1,k)}},\underset{k=n,n+1,\ldots ,n+5}{\underset{︸}{f(m,k)}},\underset{k=n,n+1,\ldots ,n+5}{\underset{︸}{f(m+1,k)}}],\sigma_{2}=[\underset{k=n+3,n+4}{\underset{︸}{f(m-3,k)}},\underset{k=n+2,n+3,n+4}{\underset{︸}{f(m-2,k)}},\underset{k=n+1,\ldots ,n+5}{\underset{︸}{f(m-1,k)}},\underset{k=n,n+1,\ldots ,n+5}{\underset{︸}{f(m,k)}}]\\ \sigma_{3}=[\underset{l=m-3,\ldots m;}{\underset{︸}{f(l,n)}}\;,\underset{l=m-3,\ldots m;}{\underset{︸}{f(l,n+1)}}\;,\underset{l=m-3,\ldots m;}{\underset{︸}{f(l,n+2)}}\;,\underset{l=m-3,\ldots m;}{\underset{︸}{f(l,n+3)}}\;,f(m,n-1),f(m-3,n+4)]\\ \sigma_{4}=[\underset{l=m-3,m-4}{\underset{︸}{f(l,n+3)}},\underset{l=m-2,m-3,m-4}{\underset{︸}{f(l,n+2)}},\underset{l=m-1,\ldots ,m-5}{\underset{︸}{f(l,n+1)}},\underset{l=m,m-1,\ldots ,m-5}{\underset{︸}{f(l,n)}}\;],\sigma_{5}=[\underset{l=m,m-1,\ldots ,m-5}{\underset{︸}{f(l,n-1)}},\underset{l=m,m-1,\ldots ,m-5}{\underset{︸}{f(l,n)}},\underset{l=m,m-1,\ldots ,m-5}{\underset{︸}{f(l,n+1)}}]\\ \sigma_{6}=[\underset{l=m-3,m-4}{\underset{︸}{f(l,n-3)}},\underset{l=m-2,m-3,m-4}{\underset{︸}{f(l,n-2)}},\underset{l=m-1,\ldots ,m-5}{\underset{︸}{f(l,n-1)}},\underset{l=m,m-1,\ldots ,m-5}{\underset{︸}{f(l,n)}}]\\ \sigma_{7}=[\underset{l=m-3,\ldots m;}{\underset{︸}{f(l,n)}}\;,\underset{l=m-3,\ldots m;}{\underset{︸}{f(l,n-1)}}\;,\underset{l=m-3,\ldots m;}{\underset{︸}{f(l,n-2)}}\;,\underset{l=m-3,\ldots m;}{\underset{︸}{f(l,n-3)}}\;,f(m,n+1),f(m-3,n-4)]\\ \sigma_{8}=[\underset{k=n-3,n-4}{\underset{︸}{f(m-3,k)}},\underset{k=n-2,n-3,n-4}{\underset{︸}{f(m-2,k)}},\underset{k=n-1,\ldots ,n-5}{\underset{︸}{f(m-1,k)}},\underset{k=n,n-1,\ldots ,n-5}{\underset{︸}{f(m,k)}}\;],\sigma_{9}=[\underset{k=n,n-1,\ldots ,n-5}{\underset{︸}{f(m-1,k)}},\underset{k=n,n-1,\ldots ,n-5}{\underset{︸}{f(m,k)}},\underset{k=n,n-1,\ldots ,n-5}{\underset{︸}{f(m+1,k)}}]\\ \sigma_{10}=[\underset{k=n-3,n-4}{\underset{︸}{f(m+3,k)}},\underset{k=n-2,n-3,n-4}{\underset{︸}{f(m+2,k)}},\underset{k=n-1,\ldots ,n-5}{\underset{︸}{f(m+1,k)}},\underset{k=n,n-1,\ldots ,n-5}{\underset{︸}{f(m,k)}}]\\ \sigma_{11}=[\underset{l=m+3,\ldots m;}{\underset{︸}{f(l,n)}}\;,\underset{l=m+3,\ldots m;}{\underset{︸}{f(l,n-1)}}\;,\underset{l=m+3,\ldots m;}{\underset{︸}{f(l,n-2)}}\;,\underset{l=m+3,\ldots m;}{\underset{︸}{f(l,n-3)}}\;,f(m,n+1),f(m+3,n-4)]\\ \sigma_{12}=[\underset{l=m+3,m+4}{\underset{︸}{f(l,n-3)}},\underset{l=m+2,m+3,m+4}{\underset{︸}{f(l,n-2)}},\underset{l=m+1,\ldots ,m+5}{\underset{︸}{f(l,n-1)}},\underset{l=m,m+1,\ldots ,m+5}{\underset{︸}{f(l,n)}}\;],\sigma_{13}=[\underset{l=m,m+1,\ldots ,m+5}{\underset{︸}{f(l,n-1)}},\underset{l=m,m+1,\ldots ,m+5}{\underset{︸}{f(l,n)}},\underset{l=m,m+1,\ldots ,m+5}{\underset{︸}{f(l,n+1)}}]\\ \sigma_{14}=[\underset{l=m+3,m+4}{\underset{︸}{f(l,n+3)}},\underset{l=m+2,m+3,m+4}{\underset{︸}{f(l,n+2)}},\underset{l=m+1,\ldots ,m+5}{\underset{︸}{f(l,n+1)}},\underset{l=m,m+1,\ldots ,m+5}{\underset{︸}{f(l,n)}}]\\ \sigma_{15}=[\underset{l=m+3,\ldots m;}{\underset{︸}{f(l,n)}}\;,\underset{l=m+3,\ldots m;}{\underset{︸}{f(l,n+1)}}\;,\underset{l=m+3,\ldots m;}{\underset{︸}{f(l,n+2)}}\;,\underset{l=m+3,\ldots m;}{\underset{︸}{f(l,n+3)}}\;,f(m,n-1),f(m+3,n+4)]\\ \sigma_{16}=[\underset{k=n+3,n+4}{\underset{︸}{f(m+3,k)}},\underset{k=n+2,n+3,n+4}{\underset{︸}{f(m+2,k)}},\underset{k=n+1,\ldots ,n+5}{\underset{︸}{f(m+1,k)}},\underset{k=n,n+1,\ldots ,n+5}{\underset{︸}{f(m,k)}}] \end{array}\right.$$

where *m* and *n* represent the horizontal and vertical coordinates of the reference point in the image, respectively. *f*(*m*, *n*) denotes the pixel value of (*m*, *n*). *σ_b_* (*b* = 1, 2, 3, …, 16) is the pixel set of the h-th sub-domain.

As can be observed in Figure 12, in an 11 × 11 filtering window, 16 sub-domains are obtained by scanning the sub-domains centered on the reference point in a counterclockwise direction, with a step of 22.5°. The scanning trajectory forms a hexadecagon approximately equivalent to a circle with a diameter of 11 pixels. The process of the modified SSFM is presented as follows.

Firstly, in each scanned sub-domain, the standard deviation *σ_s_* of the values of all image pixels within the sub-domain filtering window is calculated by using the expression written as

(5) $$\sigma_{s}=\sqrt{\frac{1}{M}\sum_{i=1}^{M}{\left(p_{i}-p_{a}\right)}^{2}}$$

where *p_i_* denotes the value of every pixel included in the sub-domain filter window, whilst *p_a_* is the average of all the pixel values in the sub-domain filter window.

Secondly, after scanning all the sub-domains, the maximum standard deviation value *σ_smax_* is extracted from the 16 calculated *σ_s_* values by using Equation (5). It should be pointed out that the angle corresponding to the sub-domain filter window with *σ_smax_* is defined as *θ*.

Next, *σ_smax_* is employed to replace the pixel value of the reference point.

Finally, the reference point of the sub-domain filtering window is moved sequentially to the next point.

**Figure 13:**
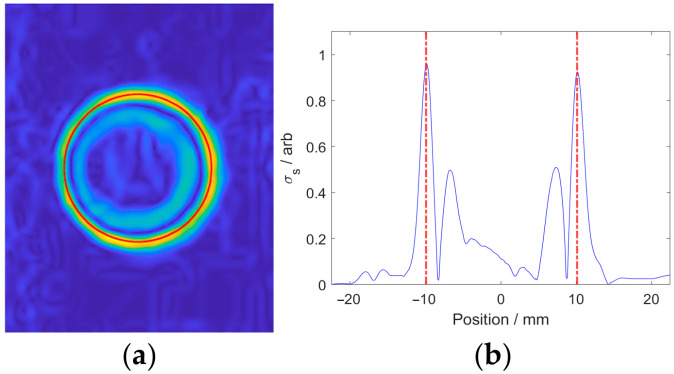
Processed results of green channel corresponding to Corrosion #4 by the improved SSFM: (**a**) processed image; (**b**) curve at the symmetry axis.

The green-channel image of Corrosion #4 processed by the improved SSFM by setting *σ* as 5 is shown in Figure 10, together with the curve composed of all pixel points at the symmetry axis. It can be seen from Figure 13 that the local pixel value maxima appear at the corrosion edge. Therefore, by retaining the edge pixels via non-maximum suppression, the accuracy of edge detection can be improved.

(3) As an edge-thinning method, non-maximum suppression is employed to produce a cleaner representation of the actual edges by reducing the number of spurious edge pixels after calculating the *σ_smax_* and *θ* of all the pixels.

To enhance computational efficiency, all pixels in the processed image are normalized. Based on the characteristics of the processed image, pixels with normalized values of less than 0.5 are directly set to 0, thereby reducing the number of pixels to be processed by at least 80%. The rest of the pixels are compared with the adjacent pixels in the angular direction. The pixel is preserved under the condition that the normalized value of the pixel is the largest among its neighbors. Otherwise, the pixel value is set to 0. Pixels with a value of 0 are barely considered as edge pixels and are, thus, removed from the processing.

(4) In spite of the processing with non-maximum suppression, a number of spurious edge pixels still remain in the image. In light of this, the edge pixels are classified into strong edge pixels, weak edge pixels and spurious edge pixels via a dual thresholding by setting a high threshold (*T_h_*) and a low threshold (*T_l_*). The edge pixels with a normalized value larger than *T_h_* are regarded as strong edge pixels (valid edge pixels), while those with a normalized value smaller than *T_l_* are deemed spurious edge pixels. The rest of the edge pixels are taken as weak edge pixels.

The valid edge pixels are extended by tracing adjacent weak edge pixels starting from each strong edge pixel. A weak edge pixel is considered a strong edge pixel provided that it is adjacent to a strong edge pixel. Otherwise, the weak edge pixel is discarded.

**Figure 14:**
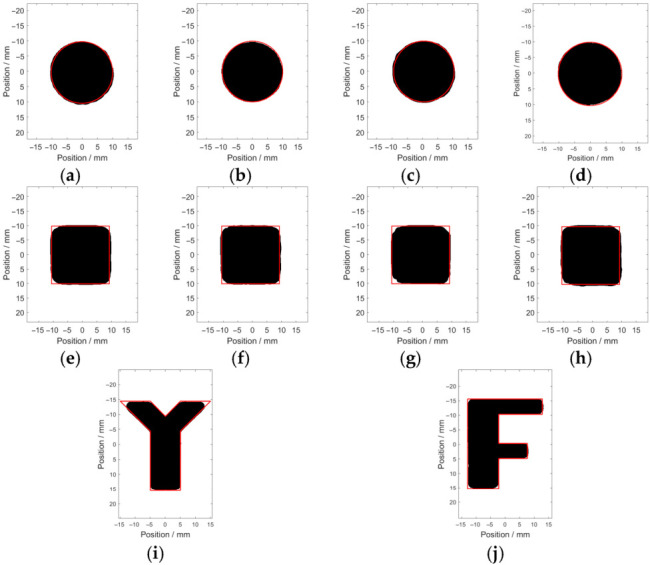
The recognition results of the hidden corrosion via the modified Canny algorithm: (**a**) Corrosion #1; (**b**) Corrosion #2; (**c**) Corrosion #3; (**d**) Corrosion #4; (**e**) Corrosion #5; (**f**) Corrosion #6; (**g**) Corrosion #7; (**h**) Corrosion #8; (**i**) Corrosion #Y; and (**j**) Corrosion #F.

The corrosion edges are recognized via the modified Canny algorithm, with *σ*, *T_h_* and *T_l_* set as 5, 0.4 and 0.8, respectively. The recognition results regarding the opening profile of each corrosion are presented in Figure 14.

It can be observed from Figure 14 that the recognized opening profile (indicated by the black area) is in good agreement with the real profile (indicated by the red solid line). Since the fabricated corrosion was milled with a milling cutter of a 3 mm radius, the convex acute angles, convex right angles and convex obtuse angles in the regular-shaped corrosion and irregular-shaped corrosion are identified as arc-shaped corners. The estimated areas of the recognized opening profile and the real area are listed in Table 4.

It can be seen from Table 4 that compared with the real area of the corrosion opening profile, the relative error of the estimated value is less than 7.4%. The average relative error is 3.95%. Particularly for the regular-shaped corrosion and irregular-shaped corrosion, the calculated average relative errors are 4.35% and 2.35%, respectively. This indicates that the accuracy of the modified Canny algorithm for identifying the corrosion opening profile is barely limited by the shape of the corrosion. As a result, the modified Canny algorithm would be feasible and capable of identifying the corrosion opening profile in engineering applications.

## 4. Evaluation of Depth and Volume of Hidden Corrosion

The high-accuracy identification of the corrosion opening profile opens a door for further quantitative assessment regarding the corrosion depth and volume. As can be observed in Figure 8, the image pixels tend to be more reddish as the depth of either the square-shaped corrosion or the circle-shaped corrosion increases. In order to investigate the correlation between the pixel value and corrosion depth, the average pixel value within the identified opening profiles via the red channel of the raw image is calculated. The computed results are listed in Table 5.

It can be seen from Table 5 that (1) the average pixel value is directly proportional to the corrosion depth; (2) there is little variation in the average pixel values corresponding to corrosions with the same depth but various shapes; and (3) there is a significant difference of approximately 30 in the average pixel values corresponding to the corrosions with depths of 1 mm and 4 mm. Consequently, the corrosion depth could be assessed via calibration based on the average pixel values across different corrosion depths.

**Figure 15:**
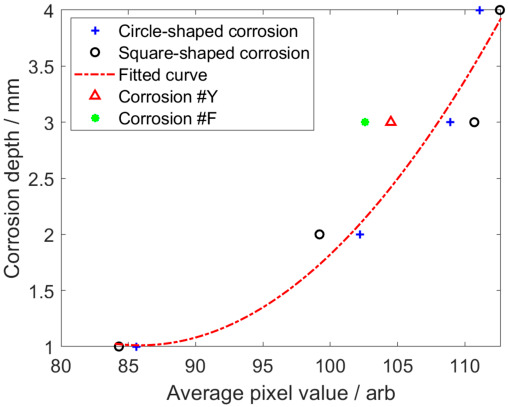
The calibration curve for square-shaped corrosion and circle-shaped corrosion.

The average pixel values of the square-shaped and circle-shaped corrosions at the same depth (1 mm, 2 mm and 4 mm) are calculated and taken as an image feature. By taking the quadratic fitting of the image features, the calibration curve in a quadratic function can be obtained. The function corresponding to the fitted calibration curve is formulated as

(6) $$h=-0.0041s^{2}-0.6964s+30.91$$

where *s* denotes the image feature, whilst *h* is the corrosion depth. The calibration curve calculated by using Equation (6) is shown in Figure 15.

By using the calibration curve for depth evaluation regarding Corrosion #3, Corrosion #7, Corrosion #Y and Corrosion #F, the approximated corrosion depths are tabulated in Table 6.

As observed in Table 6, the relative errors of the estimated depths regarding Corrosion #3 and Corrosion #7 are 5.4% and 16.7%, respectively. Both of them are less than the relative error of each irregular-shaped corrosion. This is owing to the fact that the perturbation of the magnetic field by the irregular-shaped corrosion differs from that of the regular-shaped corrosion. The estimated corrosion volumes, calculated by multiplying the estimated corrosion opening profiles and depths, are shown in Table 7.

It can be seen from Table 7 that the relative error of the estimated volume for the regular-shaped corrosion is less than 18.3%, while that for the irregular-shaped corrosion is relatively larger, i.e., up to 18.7%. The reason is that the relative error in the estimated area for the irregular-shaped corrosion is relatively larger. This indicates that the proposed MFVF-based probe, together with the modified Canny algorithm, is capable of efficiently evaluating the sizing parameters of the hidden corrosion in the CFC in terms of the opening profile as well as its area, depth and volume.

## 5. Conclusions

In this paper, the imaging and evaluation of hidden corrosion in a CFC via an MFVF-based probe were intensively investigated. The basic working principle of the MFVF-based probe was elaborated, and the feasibility of the MFVF-based probe for imaging and quantitative assessment of hidden corrosion in CFCs was verified through finite element simulation. An inspection system was built alongside specimens fabricated with regular-shaped and irregular-shaped corrosions for the experimental study. Through experiments, the methods for MFVF image processing, corrosion edge identification and assessment of corrosion depth and volume were proposed. The following concluding remarks can be drawn:

1. The magnetic field distribution of the probe was analyzed through finite element simulation, and the direction of the magnetic poles of the magnet was determined.
2. The probe rapidly images a detection area of 45 mm × 50 mm within less than 0.1 s. The corrosion imaging time of the detection system is primarily determined by the camera’s exposure time.
3. Aiming at the characteristics of MFVF images, a modified Canny algorithm was proposed, which replaces the gradient operator in the traditional Canny algorithm with an improved SSFM. The imaging results indicate that the accuracy of the quantitative assessment of the hidden corrosion profile can reach 96.0% by using the proposed algorithm.
4. After the acquisition of the corrosion profile, an assessment method for approximation regarding the corrosion depth and volume was proposed. The average pixel values corresponding to the square-shaped and circle-shaped corrosions in the red channel at the same depth were calculated to obtain a quadratic calibration curve. The evaluation accuracies for the depth and volume of the hidden corrosion based on the calibration curve were 82.5% and 79.9%, respectively.

## Figures and Tables

**Table 1:** Geometric parameters used in the simulations.

*L* /mm	*H* /mm	*l* /mm	*d* /mm	*h* /mm	*w* /mm	*l_o_* /mm
200	5	50	45	5	10	0.5

**Table 2:** Colors of MFVF corresponding to magnetic field intensities.

Magnetic Field Intensity/Gauss	Colors of MFVF
50~100	Orange
100~150	Yellow
150~250	Light green
250~300	Green
300~500	Dark green
600~800	Blue
900~1000	Dark blue

**Table 3:** Standard deviations of channels.

Channel	Red	Green	Blue
Standard deviation	0.9560	0.2751	0.8644

**Table 4:** Estimated corrosion areas and their related errors against true values.

Corrosion Scenarios	Real Area/mm^2^	Estimated Area/mm^2^	Related Error/%
Corrosion #1	314	334	6.4
Corrosion #2	314	317	1.0
Corrosion #3	314	335	6.7
Corrosion #4	314	320	1.9
Corrosion #5	392	414	5.6
Corrosion #6	392	411	4.8
Corrosion #7	392	396	1.0
Corrosion #8	392	421	7.4
Corrosion #Y	371	374	0.8
Corrosion #F	411	395	3.9

**Table 5:** Average pixel values within the identified opening profiles.

Corrosion Scenarios	Real Depth/mm	Average Pixel Value
Corrosion #1	1	85.6
Corrosion #2	2	102.2
Corrosion #3	3	108.9
Corrosion #4	4	111.1
Corrosion #5	1	84.3
Corrosion #6	2	99.2
Corrosion #7	3	110.7
Corrosion #8	4	112.7
Corrosion #Y	3	104.5
Corrosion #F	3	102.6

**Table 6:** Estimated corrosion depths and their related errors against true values.

Corrosion Scenarios	Real Depth/mm	Estimated Depth/mm	Related Error/%
Corrosion #3	3	3.162	5.4
Corrosion #7	3	3.512	16.7
Corrosion #Y	3	2.419	19.4
Corrosion #F	3	2.146	28.4

**Table 7:** Estimated corrosion volumes and their related errors against true values.

Corrosion Scenario	Real Volume/mm^3^	Estimated Volume/mm^3^	Related Error/%
Corrosion #3	942	1059	12.4
Corrosion #7	1176	1391	18.3
Corrosion #Y	1113	905	18.7
Corrosion #F	1233	848	31.2

## Data Availability

The data presented in this study are available upon request from the corresponding author after obtaining permission from an authorized person.
